# Targeting Non-coding RNA in Vascular Biology and Disease

**DOI:** 10.3389/fphys.2018.01655

**Published:** 2018-11-22

**Authors:** John Hung, Vladislav Miscianinov, Judith C. Sluimer, David E. Newby, Andrew H. Baker

**Affiliations:** ^1^Centre for Cardiovascular Science, University of Edinburgh, Edinburgh, United Kingdom; ^2^Deanery of Clinical Sciences, Centre for Cardiovascular Science, University of Edinburgh, Edinburgh, United Kingdom; ^3^Maastricht University Medical Centre, Maastricht, Netherlands

**Keywords:** vascular disease, atherosclerosis, ncRNA, microRNA, lncRNA

## Abstract

Only recently have we begun to appreciate the importance and complexity of the non-coding genome, owing in some part to truly significant advances in genomic technology such as RNA sequencing and genome-wide profiling studies. Previously thought to be non-functional transcriptional “noise,” non-coding RNAs (ncRNAs) are now known to play important roles in many diverse biological pathways, not least in vascular disease. While microRNAs (miRNA) are known to regulate protein-coding gene expression principally through mRNA degradation, long non-coding RNAs (lncRNAs) can activate and repress genes by a variety of mechanisms at both transcriptional and translational levels. These versatile molecules, with complex secondary structures, may interact with chromatin, proteins, and other RNA to form complexes with an array of functional consequences. A body of emerging evidence indicates that both classes of ncRNAs regulate multiple physiological and pathological processes in vascular physiology and disease. While dozens of miRNAs are now implicated and described in relative mechanistic depth, relatively fewer lncRNAs are well described. However, notable examples include *ANRIL*, *SMILR*, and *SENCR* in vascular smooth muscle cells; *MALAT1 and GATA-6S* in endothelial cells; and mitochondrial lncRNA *LIPCAR* as a powerful biomarker. Due to such ubiquitous involvement in pathology and well-known biogenesis and functional genetics, novel miRNA-based therapies and delivery methods are now in development, including some early stage clinical trials. Although lncRNAs may hold similar potential, much more needs to be understood about their relatively complex molecular behaviours before realistic translation into novel therapies. Here, we review the current understanding of the mechanism and function of ncRNA, focusing on miRNAs and lncRNAs in vascular disease and atherosclerosis. We discuss existing therapies and current delivery methods, emphasising the importance of miRNAs and lncRNAs as effectors and biomarkers in vascular pathology.

## Introduction

Vascular disease in its various forms remains a global health epidemic, despite decades of medical and scientific endeavour to prevent it. Cardiovascular disease alone accounts for over 17 million deaths each year, more than double what is attributable to cancer (Libby et al., 2016), and the prevalence is only expected to rise (World Health Organization [WHO], 2017). Although major advances in the fields of cardiothoracic and vascular surgery and percutaneous coronary intervention continue to improve outcomes in patients who have already suffered cardiovascular events, the key battle lies upstream in prevention. Strategies to address risk factors, such as aggressive lipid-lowering, anti-hypertensive, and anti-platelet therapies, have conferred some benefit, but vascular disease rates still continue to rise (Bhatnagar et al., 2016). Novel approaches to prevent onset and reduce progression of vascular disease are desperately needed, but progress is slow, and molecular mechanisms underpinning the pathology remain poorly understood.

Atherosclerosis is the principal driver of cardiovascular disease world-wide. It progresses over decades in the arterial walls of susceptible individuals, in response to numerous well-known risk factors, including inflammation and high circulating cholesterol. The clinical effect of atherosclerosis varies widely dependent on site, severity, and co-morbidity, and can range from completely asymptomatic to clinically catastrophic in the case of myocardial infarction (MI), stroke, and the acutely ischaemic limb. In its early stages, atherosclerosis is sub-clinical with endothelial dysfunction, intimal thickening, and plaque formation occurring silently and insidiously, only becoming clinically apparent when the plaque becomes large enough to impinge on luminal blood flow or becomes unstable and ruptures. In the coronary arteries, luminal stenosis results in angina, manifest most commonly as chest pain on exertion, and the equivalent in the peripheral vasculature, “intermittent claudication,” can be completely disabling to those affected. These chronic diseases, while not in themselves immediately life-threatening, reduce quality of life and consume large amounts of healthcare resources annually. Other sites of atherosclerosis result in chronic kidney disease and hypertension, cognitive impairment in cerebrovascular disease, and abdominal angina in mesenteric disease.

Besides lifestyle interventions, attempts at managing atherosclerosis have to date largely focused around the now well-accepted dogma that high levels of circulating lipid lead to accumulation of an expanding lipid-rich necrotic core in the sub-intimal space of the arterial wall forming predominantly in areas of low shear stress and endothelial dysfunction. Consequently, lipid lowering strategies have predominated with no less than seven different available statins, other drugs like ezetimibe, fibrates, and most recently the development of monoclonal antibodies against the PCSK9 receptor, such as evolocumab (Sabatine et al., 2017) and alirocumab (Schwartz et al., 2014). However, despite initially impressive results in the statin trials, some now doubt their efficacy in primary prevention, and staggering levels of LDL reduction as in the FOURIER trial (Sabatine et al., 2017) do not seem to translate into dramatically improved clinical outcomes. This suggests that lipid lowering may not be the only mechanism of action at play (Sabatine et al., 2017). The anti-inflammatory effect of statin drugs may be a major factor, and new drugs targeted at reducing inflammation such as canakinumab look promising. In the recently published CANTOS trial, canakinumab which targets interleukin-1β reduced the relative risk of MI in ischaemic heart disease patients by 16%, without affecting lipid levels (Ridker et al., 2017).

In the search for new therapeutically exploitable pathways, much is now known about endothelial cell (EC), smooth muscle cell (SMC), adventitial cell, and immune cell behaviour in atherosclerosis and vascular biology. The plaque is a complex environment though, and mechanistic unravelling has long been in process with notable recent additions, such as adventitial-derived mesenchymal stem cell (MSC)-like GLI1^+^ cells shown to modulate calcification in murine models (Kramann et al., 2016). EC dysfunction is the earliest known detectable abnormality in plaque formation, occurring at damage prone sites, such as vessel bifurcations. ECs entrap circulating monocytes and lipoproteins and facilitate their permeation into the sub-endothelial space (Ross and Glomset, 1976; Gimbrone and García-Cardeña, 2016). SMCs then proliferate and migrate into the intima in response to secreted chemokines and growth factors, synthesising extra-cellular matrix and sometimes undergoing phenotypic switch to macrophage-like cells, upregulating inflammation. The recruited monocytes therein become phagocytic and ingest the modified lipoproteins until they are over-laden with lipid (foam cells), and contribute to the accumulating necrotic core by undergoing apoptosis and necrosis (Ley et al., 2011; Moore et al., 2013). Although these pathways are now well investigated, there are still novel angles to interrogate, and a wealth of protein coding gene interactions still to explore. New genomic techniques such as single cell RNA sequencing (RNA-Seq) in atherosclerotic plaque promise to provide deeper insight in this respect (Tang et al., 2009; Wills et al., 2013; Cochain et al., 2018) and small interfering RNA (siRNA) therapies like inclisiran, which targets PCSK9 mRNA hold real promise (Ray et al., 2017).

This review focuses on another such avenue in the form of non-coding RNA (ncRNA). The non-coding genome represents an exciting yet complex layer in human physiology and pathology. Although some function had been previously ascribed to ncRNAs as early as the 1950s (transfer RNA and ribosomal RNA), its only in the last few decades upon the discovery that approximately 98% of the human genome is non-protein-coding (Mattick, 2001), that the potential biological significance of ncRNA has been even partly appreciated (Taft et al., 2007). MicroRNA (miRNA) is the best studied family of ncRNA, known to regulate thousands of protein-coding genes through messenger RNA (mRNA) degradation (Jonas and Izaurralde, 2015). MiRNAs are 20–25 nucleotides in length and form a characteristic “hairpin” structure. They are inherent in multiple pathologies, and due to their stability in plasma, miRNAs have also been proposed as biomarkers of MI (Bostjancic et al., 2010), cancers (Hayes et al., 2014), rheumatological, and other diseases. In terms of therapies, clinical trials are currently ongoing to determine the efficacy and safety of “antimiRs” in diseases such as cancer, hepatitis (Christopher et al., 2016), and other conditions (see later). Long non-coding RNAs (lncRNAs) are much less well characterised to date, but their importance in gene expression is increasingly being recognised. In contrast to miRNAs, lncRNAs are much longer at >200 nucleotides, with more complex secondary structures. They may act both to activate and to repress genes, exerting their effects by a variety of mechanisms at both transcriptional and translational levels (Mercer et al., 2009; Ponting et al., 2009). Evidence is limited but growing, and lncRNAs appear to be critical regulators of many processes inherent to atherosclerosis, including endothelial dysfunction, SMC behaviour, immune response, and glucose and lipid metabolism. In this review, we intend to discuss ncRNA biology and mechanisms, new and existing evidence for ncRNA in vascular disease, and the potential for delivery of novel ncRNA therapies in human vascular disease.

## Non-Coding RNA Biosynthesis and Mechanism of Action

Since their discovery in 1993 in *Caenorhabditis elegans* (Lee et al., 1993), miRNAs have now taken up an important niche in genomics with almost 2000 known sequences in the human genome. Usually, transcription of intronic miRNA is regulated by the same promoter as the host gene. However, some miRNAs, are shown to have multiple transcription start sites (Ozsolak et al., 2008) and may be transcribed by a promoter distant from the gene they occupy (Monteys et al., 2010). MiRNA transcription is controlled by RNA polymerase II and RNA Pol II-related transcription factors (Lee et al., 2004). The first product in the process is a primary miRNA (pri-miRNA) molecule, with a characteristic stem–loop structure, and lengths reaching up to 1 kilobase (kb). The next product in the sequence is a smaller precursor miRNA (pre-miRNA) molecule, which is generated by RNase III enzyme Drosha that cleaves the stem-loop of pri-miRNA (Lee et al., 2003). The small, hairpin-shaped pre-miRNA is then transported into the cytoplasm by exportin 5, where the Dicer enzyme further processes it to generate a double-stranded molecule, ready for assembly with the Argonaute (Ago) protein (Bohnsack et al., 2004; Lund et al., 2004). The final product in the miRNA biogenesis pathway is formation of the RNA-induced silencing complex (RISC), consisting of a mature, single-stranded miRNA molecule and Ago protein, which induces post-transcriptional gene silencing (Huntzinger and Izaurralde, 2011). Importantly, human miRNA lacks Ago specificity and can bind to all Ago variants (Huntzinger and Izaurralde, 2011). Recent evidence demonstrates that the Ago-unbound miRNA strand isn’t always cleaved and both the 5p and 3p strands are found in different sorts of tissues (Meijer et al., 2014). The mature miRNA then facilitates gene silencing, whereby mRNA degradation occurs in 66–90% of cases (Jonas and Izaurralde, 2015) and mechanistically depends on mRNA deadenylation, decapping, and cleavage by XRN1 nuclease (Fabian and Sonenberg, 2012). On the contrary, miRNA-induced repression of translation targets only 6–26% of genes (Eichhorn et al., 2014) using primarily RNA helicases eIF4A and DDX6, which repress cap-dependent translation (Jonas and Izaurralde, 2015).

A less simple but equally fascinating class of ncRNA is lncRNA. Remarkably, lncRNAs outnumber not only miRNAs, but also protein-coding genes. In fact, the number of lncRNAs annotated continues to rise, due to recent advances and new strategies in RNA sequencing and bioinformatic techniques, and hence a much greater depth of sequencing (Clark et al., 2015). Despite this, there is still no strict classification method, and the most accurate definition of lncRNA at present is “long RNA transcripts that do not encode proteins” (Quinn and Chang, 2016). It is generally accepted that lncRNAs should be longer than 200 nucleotides, separating them from other shorter ncRNA molecules such as miRNAs, snoRNAs, an piRNAs among others, but confusingly, some lncRNAs do actually contain cryptic open-reading frames (ORFs) (Gascoigne et al., 2012), and short open-reading frames (sORFs) encoding micropeptides. These micropeptides can act independently from large proteins, regulating essential biological processes (Makarewich and Olson, 2017). For example, a skeletal muscle-specific RNA, annotated as lncRNA LINC00948, encodes a cryptic micropeptide “myoregulin” (MLN). MLN interacts with sarcoplasmic reticulum (SR) Ca^2+^-ATPase (SERCA), preventing Ca^2+^ uptake into the SR in skeletal muscle, and accordingly MLN silencing improves Ca^2+^ handling (Anderson et al., 2015). Similarly, it was reported that lncRNA LINC00961 also encodes a polypeptide termed “small regulatory polypeptide of amino acid response” (SPAR). Mechanistically, SPAR prevents mTORC1 activation via interaction with lysosomal v-ATPase while SPAR deletion induces mTORC1 leading to muscle regeneration (Matsumoto et al., 2016). Given these clear mechanistic roles for encoded micropeptides, one might consider then that the parent lncRNA transcripts are in fact “misannotated.” How can a transcript that is experimentally proven to contain genetic code for a protein be referred to as “non-coding”? Nomenclature naturally evolves with new knowledge over time, and perhaps this will change. Clearly though there are differences between a simple mRNA which has one function only, to produce a peptide, and the complex lncRNA molecule, with its myriad of interactions and functions, which remain largely undiscovered.

Within the class of lncRNA, there are different sub-classes of lncRNAs which include long intervening/intergenic ncRNAs (lincRNAs), promoter upstream transcripts (PROMPTs), enhancer RNAs (eRNAs), and natural antisense transcripts (NATs). These are transcribed from intergenic and promoter upstream regions, enhancers, and reverse strand of protein-coding genes, respectively (Wu et al., 2017). Interestingly, lncRNAs do not require polyadenylation to be functional and upon transcription a significant proportion of lncRNA remains non-polyadenylated. In fact, many lncRNAs are bi-morphic and can exist in both polyadenylated and non-polyadenylated states (Hangauer et al., 2013). Furthermore, lncRNAs can exist in different forms and structures: some lncRNAs are capped by snoRNAs at 5′ (Wu et al., 2016) or both ends (Yin et al., 2012); others can occur in a circular form as circular intronic RNAs (ciRNAs) and circular RNA from back-splicing of exons (circRNAs) (Memczak et al., 2013; Zhang et al., 2013; Zhang X. O. et al., 2014). *ANRIL* (discussed in more detail later) is the most well-known ncRNA that can take a circular form. Currently considered to be a form of ncRNA, these transcripts exist in loops with a bond between the 3′- and 5′-ends. Much like other lncRNA, they are thought to regulate gene transcription and expression, acting as sponges for miRNA, and are extremely abundant in the circulation.

**FIGURE 1:**
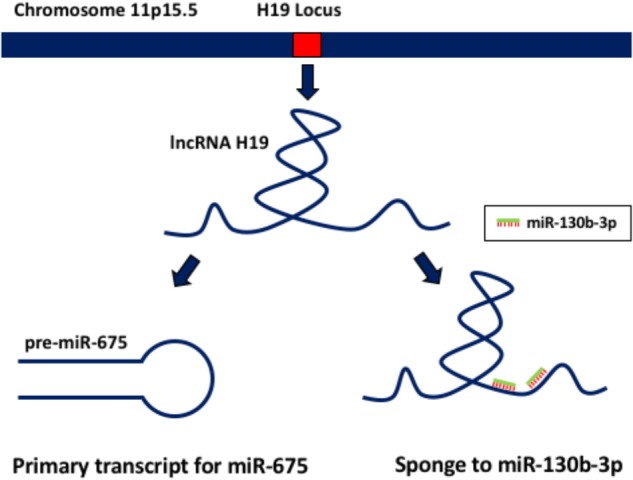
lncRNA H19 functions. This lncRNA acts both as a primary transcript giving rise to miR-675, and also as a sponge to miR-130b-3p.

Its now accepted that lncRNAs can modulate gene expression on transcriptional, post-transcriptional, and translational levels. The function of a specific lncRNA depends much on its cellular localization and context of the cell (i.e., basal/stressed). In particular, nuclear lncRNAs mainly act on transcription, while cytoplasmic lncRNAs modulate expression of gene post-transcriptionally. In the nucleus, lncRNAs regulate the epigenome, facilitate transcriptional control, and participate in alternative splicing (Zhang K. et al., 2014). A good example of lncRNA-mediated epigenetic control is *ANRIL*, which facilitates the recruitment of the chromatin-modifying complex PRC2 and promotes silencing of *p15^INK4B^* tumour suppressor gene (Kotake et al., 2010). Interestingly, after the discovery of eRNAs (Kim et al., 2010), it has been further suggested that eRNAs are able to modulate chromatin and facilitate its assembly (Mousavi et al., 2013) and interact directly with DNA structure to guide the enhancer to its promoter (Li et al., 2013). A similar type of lncRNA, termed activating ncRNAs (ncRNA-as), can also regulate transcription and are expressed from independent genes (Orom et al., 2010). Further, lncRNAs modulate different aspects of transcriptional control ranging from modulating the expression of transcription factors, such as *ncRNA-a7* (Orom et al., 2010) and *OCT4 pseudogene 5* (Bai et al., 2015), to acting as co-activators and repressors independently, such as Alu RNA inhibiting RNA polymerase Pol II directly (Mariner et al., 2008). *MALAT1* is an example of lncRNA modulation of alternative splicing. Specifically, *MALAT1* is enriched in nuclear speckles and upon interaction with SR splicing factors promotes alternative splicing (Tripathi et al., 2010). Within the cytoplasm of a cell, lncRNAs can stabilise or lead to mRNA decay and promote/inhibit translation. They can also act as miRNA precursors or sponges, mimicking mRNA for miRNA binding (Zhang K. et al., 2014). In the main lncRNA-induced mRNA degradation occurs via Staufen1 (STAU1)-mediated mRNA decay (SMD), where STAU1 binds to the pairing of mRNA 3′-UTR sequences with lncRNA due to complementary Alu elements (Park and Maquat, 2013). *BACE1-AS*, a natural antisense lncRNA forms a bond with mRNA of *BACE1* and protects it from miR-485-5p-induced degradation by masking the miRNA-binding site (Faghihi et al., 2010). LncRNA such as *Uchl1-AS* can recruit ribosomes to *Uchl1* mRNA and thus facilitate protein translation (Carrieri et al., 2012). An interesting subset of lncRNAs termed translational regulatory lncRNA (treRNA) can even repress translation. TreRNA facilitates the assembly of a new ribonucleoprotein (RNP) complex, which interacts with translation initiation factor eIF4G1 resulting in translational repression of E-cadherin (Gumireddy et al., 2013). Further, a well-studied lncRNA in cancers, *H19*, has been recently demonstrated to be a precursor for miR-675, which targets a tumour suppressor *RB* (Tsang et al., 2010). H19 is considered to be an effectual molecule itself, rather than just the primary transcript giving rise to miR-675. In the setting of keratinocyte differentiation H19 acts as a sponge to miR-130b-3p, its target (Li et al., 2017; Figure 1). Finally, it has been reported that circRNA *CDR1-as* contains 63 binding sites for miR-7 and can act a “sponge” or decoy for miR-7, thus reactivating the expression of its target genes in neuronal tissues (Hansen et al., 2013; Memczak et al., 2013).

Overall, it is clear that small ncRNAs such as miRNAs and different subsets of lncRNAs form complex molecular networks within the cell, where both classes interact closely to regulate vital cellular processes.

## Non-Coding RNA in Vascular Disease

Discovery of novel ncRNAs in disease-specific context continues to increase, largely due to the widespread application of high-throughput gene expression arrays and development of next-generation RNA sequencing. By far the most studied field in this area is cancer, but already a significant number of miRNAs are implicated in vascular disease and biology, and although much less studied, several lncRNAs have been discovered and are currently being investigated. Development, cellular differentiation, and commitment are a logical starting point to identify novel RNA candidates, and evidence of EC growth and phenotype regulation in the case of *MALAT1* increases the likelihood of an important function in pathology for this lncRNA. The scope of this review, however, focuses on ncRNAs in pathology. Here we describe some of the most important discoveries to date, adding weight to the proposition that miRNA and lncRNA transcripts represent worthwhile and plausible targets for novel gene therapies in vascular disease (see Table 1 for summary of key ncRNA in vascular diseases).

**Table 1:** Non-coding RNA in vascular disease.

Type	Vascular examples	Biological context	Reference
miRNA	miR-21	Biomarker of coronary artery disease, upregulated in vein grafts	(Raitoharju et al., 2011; McDonald et al., 2013; Han et al., 2015)
	miR-126	Promotes EC proliferation, atheroprotective	(Wang et al., 2008; Schober et al., 2014)
	miR-92a	Endothelial inflammation, atherogenic	(Loyer et al., 2014)
	miR-33a/b	Inhibits *ABCA1* translation, atherogenic	(Rayner et al., 2010; Horie et al., 2012)
	miR-143/145	Complex interaction in atherosclerosis and pulmonary hypertension: upregulated in human unstable carotid plaque, knockout blocks pulmonary hypertension in murine model	(Cipollone et al., 2011; Deng et al., 2015)
	miR-221/222	Dysregulated in acute plaque	(Cipollone et al., 2011; Maitrias et al., 2015)
	miR-1	Biomarkers for MI	(Wang et al., 2010; McManus and Ambros, 2011)
	miR-133a		
	miR-499		
	miR-208a		
	miR-192	Predictive of heart failure post-MI	(Matsumoto et al., 2013)
	miR-194		
	miR-34a		
lncRNA	ANRIL	Transcribed from 9p21 locus, associated with pathogenic changes in atherosclerotic plaques	(Holdt et al., 2010)
	MIAT	Biomarkers for MI	(Ishii et al., 2006; Vausort et al., 2014; Zangrando et al., 2014)
	MIRT1/2		
	HIF1-AS2		
	KCNQ1OT1		
	SENCR	Downregulated in human critical limb ischaemia, and in premature coronary artery disease	(Boulberdaa et al., 2016)
	SMILR	Induces SMC proliferation, upregulated in human carotid plaques	(Ballantyne et al., 2016)
	meXis	Improves cholesterol efflux, atheroprotective	(Sallam et al., 2018)
	MALAT1	Downregulated in plaque, endothelial phenotypic switch	(Arslan et al., 2017)
	LIPCAR	Predictive of heart failure post-MI	(Kumarswamy et al., 2014)


## MiRNAs

There is a large body of evidence demonstrating miRNA involvement in many of the pathological processes that occur in vascular disease, and atherosclerosis. Hundreds of miRNAs are now reported as key regulators of lipid handling, inflammation, and cellular behaviours, such as SMC and EC proliferation, migration, and phenotypic switch. Translation of these important mechanistic findings into novel therapies in humans involves a long and expensive process, and the vast majority of mechanistic investigation is undertaken pre-clinically, *in vitro*, and in animal models. Human experimental evidence usually amounts to correlative findings in excised tissue samples, such as carotid plaque, or in readily available plasma or cellular blood components like peripheral blood mononuclear cells (PBMCs).

As a readily accessible source of human tissue, carotid plaque profiling has led to discovery and validation of several dysregulated ncRNAs in this way. MiR-21 for example is an important miRNA, which was found to be increased more than sevenfold in human peripheral arteries with severe atherosclerotic disease compared with controls. MiR-21 was further shown to be increased in the circulation of *apoE* knockout mice and humans with coronary artery disease (Raitoharju et al., 2011; Han et al., 2015). In clinical application, expression of miR-21 was shown to rise after engraftment of veins into murine and porcine models, and in cultured human saphenous vein explants. Subsequent knockdown in *ex vivo* human saphenous vein explants using anti-miRs was then >95% efficacious, and neointima formation was attenuated (McDonald et al., 2013). MiRNAs can be tissue and disease specific, but as in the case of miR-21, some transcripts are enriched across multiple tissues, and regulate multiple processes (Ludwig et al., 2016). While known to be an important factor in fibrosis and neointima formation in vascular disease as above, miR-21 is also thought to be part of an inflammatory positive feedback loop in vascular ECs. It promotes adherence of monocytes via activator protein AP-1, as well as negatively regulating LPS-induced lipid accumulation and inflammatory responses in macrophages by the TLR4–NF-κB pathway (Feng et al., 2014). The well-expressed and widespread miR-21 has in fact now been targeted in several other diseases too, including kidney disease, and cancer and trials of therapy are well underway (Denby et al., 2014; Nedaeinia et al., 2017).

Endothelial cell dysfunction is a critical and probable triggering event in vascular disease mediated at least in part by disturbed laminar flow in predisposed locations such as vessel bifurcations. MiR-126 is an EC-enriched miRNA (Wang et al., 2008) and is critical for EC proliferative reserve in such areas, effective by suppression of the Notch1 inhibitor delta-like 1 homolog (Dlk1). When denuded, recovery of the endothelial layer was compromised when miR-126-5p deficient, and treatment with miR-126-5p could rescue proliferation and thereafter protect against atherosclerosis, suggesting a possible therapeutic angle for this miR (Schober et al., 2014). Investigating the effect of blood flow on miR expression, pulsatile flow was shown to increase expression of the well-studied miR-92a (while laminar flow reduces it) (Wu et al., 2011; Fang and Davies, 2012) via the regulation of Kruppel-like factor 2 (KLF2), which is a positive regulator of nitric oxide synthesis. MiR-92a inhibition then reduced endothelial inflammation, and hence plaque size (Loyer et al., 2014). Clinical development of an “antagomir” designed to inhibit miR-92a is now in process, and multiple other animal models have demonstrated significant vascular benefit in downregulating this important transcript. Roles have since been defined beyond endothelial dysfunction too, in angiogenesis and proliferation, translating into enhanced cardiac recovery and cardiac protection (see later).

Lipid handing is also central to evolution of atherosclerotic plaques and appears to be regulated by a number of different miRNAs. The adenosine triphosphate-binding cassette (ABC) transporter, *ABCA*1 specifically is subjected to complex miRNA regulation, and ABCA1 is known to be critical to cholesterol transport and high-density lipoprotein (HDL) biogenesis in the liver (Rotllan et al., 2016). This is particularly important because HDL is recognised as one of the few reliably validated risk factors in atherosclerotic disease. There have been multiple unsuccessful pharmacological attempts to increase HDL. Trials using niacin and *CEPTB* inhibitors, such as torcetrapib, have shown no benefit and in fact, the ILLUMINATE trial was stopped early due an increase in all cause mortality in the treatment group (Barter et al., 2007; Boden et al., 2011). MiRNA manipulation may still be possible though, as we know that *ABCA1* translation is directly inhibited by miR-33 (Rayner et al., 2010) which results in attenuation of cholesterol efflux, and when miR-33 is genetically deleted in atherogenic murine models, plaque volume and lipid content are reduced (Horie et al., 2012). Correspondingly, circulating HDL is increased in miR-33 knockout mice, and reduced when it is overexpressed. This has been further validated in non-human primates, perhaps more reassuring of significance in humans, particularly as the mouse locus does not encode miR-33b, whereas in primates the local genomic profile is much more similar (Rayner et al., 2011). MiR-33a and miR-33b remain exciting targets in vascular prevention, but recent evidence suggests that while loss of these miRNA may be beneficial in terms of macrophage behaviour within the plaque (reduced size and volume), full knockout actually results in obesity, insulin resistance, and hyperlipidaemia overall, albeit in a mouse model (Price et al., 2017). Obviously the environment is complex, and the *ABCA1* locus is regulated by a number of other miRNAs, and interestingly a recently discovered lncRNA *meXis* (Rottiers and Näär, 2012; Sun et al., 2012; Zhang M. et al., 2014; Sallam et al., 2018) (described later).

In vascular SMCs, the miR-143/145 cluster is now a well-recognised regulator of cell function. Generally, the literature describes downregulation in the disease state, and an improvement when replaced or over-expressed. For example, in murine aortic constriction, miR-143/145 expression is reduced, and knockout mice have abnormal arterial structures and evidence of de-differentiation on histological analysis (Elia et al., 2009). Further, the knock-out of *miR-143/145* resulted in loss of SMC contractility and favoured neointimal formation (Boettger et al., 2009). Whereas in lentiviral overexpression of miR-145, plaque appearances were overall improved with a reduction in plaque size, and amelioration of features associated with instability such as size of lipid core and thickness of fibrous cap (Lovren et al., 2012). Findings in human samples then largely corroborate the hypothesis that the cluster is downregulated in disease, consistent in aortic aneurysm tissue versus control, and in the PBMCs of hypertensive patients (Elia et al., 2009; Kontaraki et al., 2014). It has been demonstrated that the process of actin depolymerisation in mild aortic dilation leads to downregulation of miR-143/145 regulated by myocardin-related transcription factors (MRTFs) (Alajbegovic et al., 2017). However, another level of complexity is suggested by conflicting evidence in apparent upregulation in carotid artery plaques of patients with stroke (Cipollone et al., 2011), and similarly higher expression in the carotid plaques of patients with hypertension (Santovito et al., 2013), and pulmonary artery SMCs of patients with pulmonary hypertension (Caruso et al., 2012; Deng et al., 2015). Clearly much is yet to be learned about their significance, and complex interactions before precise therapeutic options are defined in this case.

The miR-221/222 cluster is another important regulator of vascular cell function in plaque rupture although it too has a complicated, sometimes conflicting representation in the literature. Prevention of plaque rupture is of course an important aim of treatment in vascular disease, whether carotid, coronary, or peripheral. The miR-221/222 cluster has been implicated in this acute process, and while consistently identified as a dysregulated miRNA in acute plaque (Cipollone et al., 2011; Maitrias et al., 2015) was shown to have apparently opposing effects dependent on cell type in VSMCs and ECs (pro-proliferative/migratory in VSMCs, yet anti-proliferative/migratory in ECs) (Liu et al., 2012). Critically, miR-221/222 levels appear to reduce at the time of plaque rupture, and then normalise around 2 weeks after the event (Bazan et al., 2015). Precision targeting of this cluster in the plaque to prevent fibrous cap degradation, hence, seems beneficial. However, the widespread effects of manipulating circulating levels of miR-221/222 need to be thoroughly studied, because this very well-conserved miRNA appears to be enriched in patients with diabetes (Coleman et al., 2013) and plays important roles in various types of cancer, too (Song J. et al., 2017). As with any ncRNA therapy targeted at a particular disease process in specific cells or tissues, the specificity of action is hence absolutely critical. Similarly, in the development of an anti-miR to miR-21, off-target effects of systemic dissemination were observed as expected, including a rise in serum creatinine, but some success was had with a drug-eluting stent (DES) delivery which reduced in-stent restenosis, without off-target problems (Wang et al., 2015) (see later).

## LncRNAs

Increasingly, lncRNAs emanating from multiple sources are shown to be implicated in cardiovascular disease. The lncRNA anti-sense noncoding RNA in the *INK4* locus (*ANRIL*) is transcribed from the now well-known 9p21 locus, which has been strongly implicated in vascular disease (Holdt et al., 2010; Congrains et al., 2012; Cheng et al., 2017). It was initially discovered through genome-wide association studies detecting several polymorphisms, which were predictive of atherosclerosis (Wellcome Trust Case Control Consortium, 2007; Samani et al., 2007). Notably, important tumour suppressor genes *CDKN2A* and *CDKN2B* are also found near this region, but are not found to be dysregulated in atherosclerosis models and patients, unlike *ANRIL* (Chen et al., 2014). *ANRIL* was one of the first lncRNAs described in atherosclerosis, and multiple interactions have now been demonstrated, although the full extent of its regulation in vascular disease remains unclear. As is the case with most lncRNAs, *ANRIL* is alternatively spliced, and expressed as multiple “isoforms.” Ensembl currently reports 21 splice variants, each of which may interact differently to the others. Further adding to its complexity, *ANRIL* is found in multiple cell types including SMCs, ECs, and inflammatory cells. Initially, *ANRIL* appeared to be grossly associated with pathogenic changes; reduced cell viability and proliferation in SMCs (Congrains et al., 2012), upregulation of inflammation and apoptosis in ECs (Song C.L. et al., 2017), and a consistent correlation with atherosclerotic burden in human PBMCs and plaque samples (Holdt et al., 2010). More recently though a circular variant has been characterised, *circANRIL*, which seems to be atheroprotective in its behaviour; by controlling ribosomal RNA biogenesis and modulating some atherogenic processes in VSMCs and macrophages (Holdt et al., 2016). Unless otherwise stated, *ANRIL* would hence usually refer to the linear transcript (*linANRIL*). This alternatively structured RNA from the same locus exemplifies the added layers of complexity in lncRNA biology compared with smaller micro and other ncRNAs.

Until now, *in vitro* work has led to limited characterisation of some other vascular lncRNAs in relevant cell types, but translation into definite therapeutic targets in humans requires complex mechanistic unravelling, which in most cases is in the early stages. LncRNAs identified in circulatory samples from atherosclerosis patients, or from GWAS, may provide a starting point for vascular targets, but confounding processes such as myocardial injury and repair or the general upregulation of inflammation are equally likely to account for these correlative findings. Notable examples include MI-associated transcript (*MIAT*) (Ishii et al., 2006), another transcript discovered by large case–control genome association study in patients with MI, and MI-related transcripts 1 and 2 (*MIRT1*, *MIRT2*) in a murine MI model (Zangrando et al., 2014). While dysregulation in the disease state is apparent, mechanistic work to show why this is remains to be seen.

As with miRNAs, a certain insight has been achieved from *in vitro* cellular models. Although cultured cell lines representing one cell type are not truly analogous to the multicellular *in vivo* environment, it is possible to investigate candidate novel transcripts, and to identify at least if these are important in cellular function in the first instance. In coronary artery SMCs, the smooth muscle and EC-enriched migration/differentiation-associated lncRNA (*SENCR*) is an example of a transcript discovered by RNA sequencing, and functionally characterised in relevant cell types. *SENCR* was shown to be anti-migratory in SMCs, and subsequent RNA-Seq after *SENCR* knockdown then demonstrated a reduced expression of myocardin and numerous contractile genes, consistent with the observed phenotype (Bell et al., 2014). Subsequently, lower levels of *SENCR* in human critical limb ischemia and in the ECs of patients with premature coronary artery disease were observed (Boulberdaa et al., 2016), again supporting that hypothesis that *SENCR* is downregulated in pathology. One might propose then that targeted delivery of *SENCR* in areas of deficiency might then ameliorate maladaptive SMC behaviours. Another vascular lncRNA, smooth-muscle-induced lncRNA (*SMILR*), was investigated in a similar manner, but in this case upregulation of *SMILR* was associated with vascular SMC proliferation, and in human atherosclerotic plaque *SMILR* is enriched. Mechanistic investigation showed that *SMILR* may exert its effects by interaction with *cis* protein *HAS2*, which codes for hyaluronic acid. *HAS2* was also reduced on *SMILR* knockdown, and the SMC phenotype was of reduced proliferation (Ballantyne et al., 2016). Therapeutic targeting of *SMILR* with *in vivo* locked nucleic acids (LNAs) might prove useful in preventing neointima formation in clinical settings.

Aside from the cells which are inherent to the vascular wall, inflammatory cells like monocytes and macrophages are the key regulators of lipid handling within the plaque, and recently it was shown that the lncRNA *meXis* is an amplifier of the *ABCA1* gene, via the sterol activated liver X receptors (LXRs). LXRs are sterol-activated nuclear transcription factors, and may be important in the pathology of atherosclerosis, as key regulators of genes involved in cholesterol transport. *MeXis* interacts with and guides promoter binding of the transcriptional coactivator *DDX17*, so loss of the gene resulted in impaired cholesterol efflux, and accelerated atherosclerosis in murine models. As discussed, any novel target for improving cholesterol efflux (which is beneficial in plaque disease) certainly warrants further investigation (Sallam et al., 2018).

Utilising human samples excised at coronary endarterectomy a recent study compared lncRNA expression from diseased left anterior descending (LAD) artery plaque with inferior mammary artery control tissue. This is a rarely performed procedure, and coronary artery samples from live patients are not usually readily available. The inferior mammary artery used as a control here is preferentially used for LAD coronary artery bypass and tends to be quite resistant to atherosclerosis with low failure rates (Etienne et al., 2013). Differential lncRNA expression was observed in three of the five plaques chosen for analysis, with upregulation of *ANRIL* and *MIAT* while metastasis-associated lung adenocarcinoma transcript 1 (*MALAT1*) was downregulated (Arslan et al., 2017). This reinforces the initial premise that these lncRNAs which had been previously proposed as atherosclerosis-related lncRNAs, and had been found in the circulation of patients with acute MI (Vausort et al., 2014), are in fact reproducibly found in human tissue.

*MALAT1* briefly described above is frequently cited in vascular disease and widely in multiple pathologies (primarily cancer) is a very well-conserved and highly expressed lncRNA (Gutschner et al., 2013). Having initially been demonstrated as a prognostic marker in non-small cell lung cancer (Ji et al., 2003), *MALAT1* appears to be also relevant in vascular disease, and was shown to control phenotypic switch in ECs, as well as impair vessel recovery in a hind-limb ischaemia model when knocked down using *in vivo* GapmeRs (Liu et al., 2014; Michalik et al., 2014). The use of *in vivo* GapmeRs to knock down chosen ncRNAs will be discussed later.

## Non-Coding RNA as Biomarkers

Peripheral blood samples are easy to access, as are large-scale array systems for identification of differentially regulated genes, so it is unsurprising that there are now many miRNAs described as biomarkers of various diseases. In addition, miRNAs are quite well expressed in the circulation, and stable in stored samples of blood and plasma, throughout freeze/thaw cycles, making them reasonable to handle (Chen et al., 2008; Mitchell et al., 2008).

Examples in vascular disease include striated muscle-enriched miRNAs such as miR-1, miR-133a, and miR-499, which were upregulated in a population of acute MI patients and were highly sensitive for MI by receiver operating characteristic (ROC) curve analysis (Wang et al., 2010) compared with patients with no MI. Similarly, miR-197 and miR-223 were shown to be predictive of future cardiovascular death over a 4-year follow up in a large sample of patients with documented coronary artery disease, some with and some without acute infarction (Schulte et al., 2015). These are two miRNAs which have been serially implicated in this context, and in a different study were shown to predict future cardiovascular events from baseline. This time there was a negative correlation between circulating levels of miR-197 and miR-223, and another miRNA miR-126 was shown to be positively correlated. More in depth investigation showed that miR-223 and 197 were highly expressed in platelets, whereas miR-126 was more EC enriched (Zampetaki et al., 2012). The origin of circulating transcripts is usually difficult to ascribe, but as the authors speculate in this case, it seems in-keeping with previous knowledge that the lower levels of miR-197 and miR-223 may reflect platelet dysfunction, and the higher levels of miR-126 could be indicative of endothelial dysfunction.

In heart failure post-MI, miR-192, miR-194, and miR-34a are predictive of heart failure up to 1 year, and correlate with left ventricular dilatation on echocardiography (Matsumoto et al., 2013). Centrally acquired samples from the coronary circulation are potentially more valuable though, and in a more intricate biomarker study the localisation of miRNA release was characterised in a similar panel of candidates. Using coronary catheters to access the aorta and coronary venous sinus, simultaneous EDTA blood samples were taken from patients with stable coronary disease, troponin positive acute coronary syndrome (ACS), and normal controls. Not only were miR-133a, miR-499, and miR-208a elevated in patients with ACS, but a *trans*-coronary gradient was noted in miR-133 and miR-499, strongly suggestive of myocardial or coronary release due to injury (McManus and Ambros, 2011). As well as validating the novel biomarkers in ACS, this study suggests that localised cardiac sampling may be preferable to peripheral methods, especially in lowly expressed targets. Peripheral blood sampling remains the easiest and most accessible route, however, and caution must be exercised when undertaking PCR in centrally acquired samples, because heparin which is frequently used to anticoagulated cardiac patients undergoing cardiac catheterisation is known to inhibit PCR downstream due to binding of proteins required for the reaction (Beutler et al., 1990).

Although comparatively fewer lncRNAs are described as biomarkers of vascular disease thus far, these versatile molecules have been reliably detected in the circulation in cancer (Bolha et al., 2017) and their tissue specificity like miRNA makes them likely candidates. In a cohort of 414 patients with acute MI, the expression levels of five known lncRNAs in circulating cells were measured, demonstrating higher levels of hypoxia inducible factor 1A antisense RNA 2 (*HIF1A-AS2*), *KCNQ1OT1*, and *MALAT1* compared with controls, while *ANRIL* went down. In addition, four of the five were predictive of future left ventricular systolic function. Similarly, long intergenic ncRNA predicting cardiac remodelling (*LIPCAR*) is readily detectable in human plasma and is uncharacteristically well expressed for a lncRNA. In a global transcriptomic analysis of patients post-MI, *LIPCAR* independently predicted cardiovascular death, despite being initially lower expressed immediately after MI, levels subsequently rose (Kumarswamy et al., 2014).

Already it is clear that many ncRNA species are deeply involved in the pathology of vascular diseases. These manipulable molecules represent feasible targets for therapeutic modification, but also as diagnostic biomarkers or predictors of risk. It seems unlikely that ncRNA could ever replace cardiac troponins in the evaluation of sudden onset chest pain and diagnosis of MI, which are tremendously well validated, but due to their potential specificity to individual processes within the pathology, such as plaque instability, they may still find a role (Chapman et al., 2017).

## Non-Coding RNA Limitations as Biomarkers

While ncRNA hence have the required specificity to pathology to be suitable biomarkers, a current limitation is in their extraction and quantification. Unlike highly abundant proteins such as troponin, which can be easily measured using an ELISA assay for example, RNA is more difficult to isolate in reasonable quantities and reproducibly so from acellular bodily fluids such as plasma or serum. Very small amounts of RNA are generally exported in extracellular vesicles, as in the case of miRNA, and some likely escapes from damaged circulating or vascular cells and may be bound to proteins. However, the exact origins of circulating lncRNA in particular are not at all understood, and the vast majority of RNA in the body is intracellular. However, traditional methods, such as Nanodrop^TM^ spectrophotometry, fluorometric measurement using Qbit^TM^, and even automated electrophoresis with Agilent Bioanalyzer which are commonplace for cellular RNA applications, are not sensitive enough in realistically sized sample volumes of cell-free bodily fluids. Not only does this hamper scientific study and development, but inherent biases may also be introduced. With the expected low expression of ncRNA molecules, cycle threshold (CT) values on qPCR will be very high, towards the upper limit of detection for the machinery, and become less reliable. Of course, accuracy is always compromised throughout the entire work stream when the absolute quantity is reduced. Furthermore, in the discovery phase, the tendency to study only the highest expressed of the ncRNA species leads to neglect of the less well-expressed transcripts. Lower abundance does not necessarily mean lower importance. As a result, although multiple studies report differential expression of novel lncRNA biomarkers in human plasma and serum, others have found difficulty in replicating (Schlosser et al., 2016). A noteable exception is LIPCAR, which is highly expressed and consistently detectable in plasma samples (Kumarswamy et al., 2014).

## Non-Coding RNA Therapeutics and Delivery Methods

**FIGURE 2:**
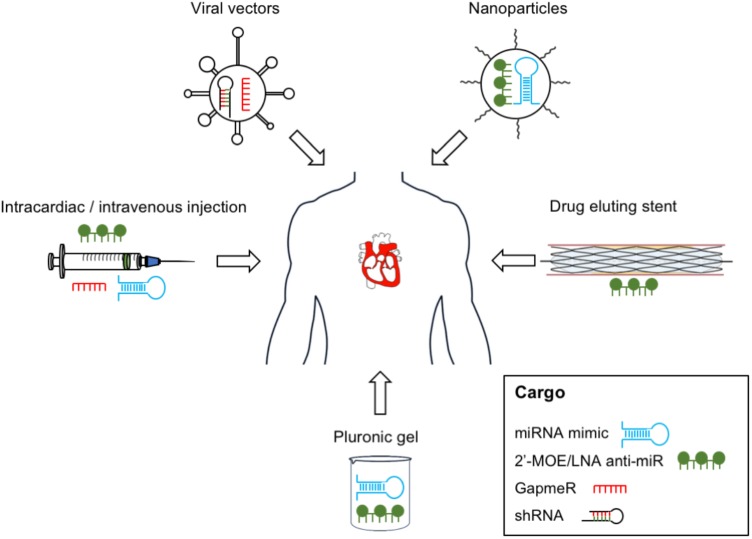
Non-coding RNA therapy delivery – proposed delivery methods of ncRNA-based therapeutics in vascular disease. RNA therapeutics can be delivered to the vascular system using vehicles such as viral vectors, nanoparticles, and pluronic gel; intravascular devices such as drug-eluting stents; or by direct injection.

Here we discuss current knowledge on new and emerging therapies using ncRNA technologies. Proposed methods of delivery are shown in Figure 2.

## MiRNA as Therapeutic Agents

In the large class of ncRNAs, miRNAs currently possess the strongest therapeutic potential due to our greater knowledge of them, clearer mode of action, and ability to regulate multiple genes in multiple molecular pathways. Moreover, due to their pleiotropic mechanism of action, a manipulation of a single miRNA could potentially induce a therapeutic effect within several cells and tissues. With re-emerging interest in RNAi therapeutics (Zeliadt, 2014), it is possible that miRNA-based therapies will progress further in the coming years.

Ultimately, efficacy depends on both modification of the RNA molecule, and its vehicle for delivery. The two principle strategies for miRNA therapeutics include overexpression of miRNA by synthetic oligoribonucleotide (ORN) delivery or targeted miRNA inhibition using single-stranded antisense oligonucleotides (anti-miRs). In order to obtain better delivery efficiency, the ORNs can be subjected to chemical modifications, such as 2′-*O*-methoxyethyl (2′-MOE) substitutions and LNA bases, which stabilise the ORNs (Hutvagner et al., 2004; Orom et al., 2006; Elmen et al., 2008a). In particular, the 2′-MOE modification is generally used to prevent degradation of ORN as well as to mask it from the immune response, whereas LNA enhances the affinity of miRNA and stabilises the ORN further (Davis et al., 2006; Henry et al., 2011). The use of such modifications to improve expression modulation of certain miRNA has been validated numerously in the vascular disease setting, including examples such as delivery of 2′-OMe-miR-21 to rat carotid artery (Ji et al., 2007), systemic downregulation of miR-92a with LNA-anti-miR to induce re-endothelialisation (Daniel et al., 2014) as well as intravenous delivery of LNA-anti-miR-15b to reduce cardiac remodelling in mice post-MI (Hullinger et al., 2012).

In addition to ORN modifications, efficient RNA delivery vehicles are required to facilitate their uptake. There are several studied and trialled thus far, as discussed below. The most common delivery method to date is the use of lipid-based nanocarriers, which package the RNA, allowing it to cross the cellular membrane (Pirollo et al., 2008; Trang et al., 2011). Notably, in a study carried out by Weber’s group it was shown that miR-126-5p mimics packaged in this way were effectively and efficiently transfected *in vivo*. The cargo was delivered intravenously to high cholesterol diet mice, resulting in a marginal decrease in atherosclerotic lesion formation, and an increase in luminal EC proliferation. They reported in the same study that using pluronic gel-based delivery was also effective, in this case for local delivery of anti-miRs injected around the mouse carotid artery (Schober et al., 2014). Pluronic gel can be used internally or topically, and was also used effectively in a recent study by Miscianinov et al., where miR-148b mimics were topically administered to efficiently enhance angiogenesis and wound healing *in vivo*. It was noted in this case, however, that the delivery of anti-miR-148b within the pluronic gel had impaired wound closure and induced EndMT in the wound vasculature, possibly a result of the target manipulation more-so than the mode of delivery (Miscianinov et al., 2018). Alternative methods to package and deliver RNA-based therapies involve polymer- and peptide-based systems, such as polylactic-co-glycolic acid (PLGA) and polyamine-co-ester terpolymer (PACE) nanoparticles. The latter are able to release the ORNs steadily over time (Woodrow et al., 2009; Zhou et al., 2011). PLGA was used in the vascular setting when an anti-miR to miR-92a encapsulated by PLGA microspheres was delivered directly to pig coronary arteries. MiR-92a expression was downregulated locally, preventing left ventricular remodelling in a model of reperfused MI (Bellera et al., 2014).

Of course, viral vectors such as adenovirus (AV) and adeno-associated virus (AAV) are among the best known vehicles for gene therapies due to their natural ability to infect, and transfer genetic information. Viruses have been used to efficiently deliver many different cargoes *in vivo* previously like the tissue inhibitor of metalloproteinase-3 (TIMP3) in vein graft failure, which reduced neointimal formation consistently (George et al., 2000). The commonly used vector AAV has been equally well-studied, and delivery of hsa-miR-590 and hsa-miR-199a pre-miRs to the neonatal rat heart was shown to significantly reduce infarct size and improve cardiac function post-MI (Eulalio et al., 2012). Despite concerted efforts to optimise this process however, viral delivery can be hampered by several drawbacks, namely the triggering of immune response (especially in the case of AV), off target effects, and the sometimes undesirable long-term incorporation of the virus’s genes into those of the host.

Specificity of site of action hence represents one of the major barriers to developing successful miRNA therapies, as in both of the above classes of examples. However, some success has been seen with attempts to deliver miRNA therapies locally. In the vasculature in particular, drug eluting stents were already widely used in percutaenous coronary intervention, releasing anti-proliferative and immunosuppressive drugs to prevent re-stenosis. In a similar approach, it was shown that delivery of LNA anti-miR-21 by DES significantly attenuated in-stent restenosis in the humanised rat myointimal hyperplasia model (Wang et al., 2015). Photoactivatable antimiRs, which have photolabile cages attached to the oligonucleotide structure are only activated by light, therefore generating an inducible model (Zheng et al., 2011; Connelly et al., 2012). This ensures that the miR treatment is only efficiently released at the intended site. Clearly this is useful in skin, but perhaps less so internally. Efficacy of a miR-92a light-inducible compound was demonstrated in superficial mice wounds, and was able to enhance proliferation and angiogenesis (Lucas et al., 2017). Finally, incorporation of miRNA therapy with a thioaptamer, specifically interacting with a chosen ligand, E-selectin in this example, may be useful to ensure specificity. The aptamer binds to the chosen molecule and guides the miR therapy, miR-146a and miR-181b to inflamed endothelium, reducing atherosclerosis in mice (Ma et al., 2016).

As there are currently no clinical trials focused on ncRNAs in vascular disease, we will describe studies in liver and heart and translational relevance for vascular disease. The most successful example of miRNA-based therapeutics to date is anti-miR-122 compound for hepatitis C. It has been demonstrated in Jopling et al. study that miR-122 is highly and specifically expressed in human liver. Moreover, it has been shown that inhibition of miR-122 was able to strongly decrease viral RNA of hepatitis C, introducing the novel idea for a potential miR-122-modulating therapy (Jopling et al., 2005). Since then a lot of work has been done to achieve an efficient miR-122 inhibition method *in vivo*, primarily using 2′-MOE-modified anti-miR (Krutzfeldt et al., 2005; Esau et al., 2006). Furthermore, anti-miR-122 LNA modification approach was efficient in downregulating miR-122 expression in mice, which was delivered intravenously (Elmen et al., 2008b). Notably, preclinical trials on chimpanzees have confirmed that LNA-anti-miR-122, now known as SPC3649 or Miravirsen, leads to significant reduction in hepatitis C viral load with no side-effects (Lanford et al., 2010). Currently, Miravirsen is being developed by Santaris Pharma and since 2017 is in Phase II clinical trials (Titze-de-Almeida et al., 2017).

In other translational studies, direct intra-cardiac injection of miR-21, miR-1, and miR-24 reduced infarct size in mice, with LAD ligation, at 24 h (Yin et al., 2009). The mice underwent ischaemic preconditioning, forcing an altered miRNA expression profile, with the hypothesis that these miRNA would be protective in the case of infarct. The miRNAs were then extracted and injected into the infarct model, resulting in an increase of eNOS, HSF-1, and HSP70. Direct injection of miRNA seems to be effective therefore in altering tissue expression of important mRNAs and proteins. In a mesenchymal stem cell vector, miR-1 was overexpressed and injected into infarcted myocardium again in a mouse model of infarct, resulting in enhanced cell survival and improved cardiac function (Huang et al., 2013). In a similar way but with a different mode of delivery, polyketal (PK3) nanoparticles (a solid polymer) were used to deliver miRNA mimics miR-106b, miR-148b, and miR-204 to macrophages in mice hearts, all targeting Nox2 expression. Infarct size was significantly reduced, and function improved (Yang et al., 2017). The alternative approach of miRNA inhibition has also been shown to be effective in the cardiovascular setting, and in some cases may be technically easier to achieve. Anti-miRs can be quickly designed and developed, and *in vivo* delivery of the anti-miR-143 is protective in the development of pulmonary arterial hypertension in mice (Deng et al., 2015). MiR-143-3p is selectively upregulated in cell migration, and its modulation significantly reduces cell migration and apoptosis.

Vein graft failure in surgical coronary artery bypass has been a longstanding problem, and the setting for some significant advances in ncRNA therapy. MiR-21 expression is elevated in mice, porcine, and human *ex vivo* models of vein graft failure, and localises to the SMC layers of the forming neointima in the failing grafts. Delivery of anti-miR-21 to inhibit miR-21 was effective to reduce expression and attenuate the pathological neointima formation in a model of vein graft failure (McDonald et al., 2013), and now further clinical investigation is needed before this can be applied clinically. In a completely different disease system its also been reported that inhibition of the same miR-21 using anti-miR treatment can prevent Alport syndrome in mice. This disease which is characterised by glomerulonephritis and progressive renal failure also results in sensorineural deafness in the human form (Gomez et al., 2015). As a result of these findings, ‘Regulus Therapeutics’ are now carrying out a Phase II clinical study (NCT02855268) of the safety and efficacy of RG-012 drug (anti-miR-21) in the treatment of patients with Alport syndrome.

In the targeting of another miRNA, miR-92a, it was shown that downregulation of miR-92a expression using 2′-*O*-methyl anti-miR ORNs enhanced *in vivo* angiogenesis, neovascularisation as well as enhanced post-MI recovery in mice (Bonauer et al., 2009). This apparent pro-angiogenic and cardioprotective effect of anti-miR-92a therapy was further investigated by the same group. Specifically, they demonstrated that catheter-based delivery of anti-miR-92a with LNA modification (LNA-92a) led to a decrease in the infarct size in pig hearts and improved cardiac function (Hinkel et al., 2013).

With the advances of CRISPR/Cas9 technology and the ability to modify the genome at the base pair level, this method has become a very attractive and promising approach to generate both gain- and loss-of-function miRNA phenotypes. Interestingly, an *in vitro* transfection of CRISPR/Cas9 vectors, which contain sgRNAs, targeting the biogenesis processing sites of miR-17, miR-200c, and miR-141, decrease the expression of these miRNAs up to 96% (Chang et al., 2016), an impressive knockdown. Moreover, subcutaneous injection of HT-29 cells with CRISPR/Cas9-mediated *miR-17* knockdown into nude mice resulted in almost complete knockout of miR-17 expression in the *in vivo* environment after 28 days (Chang et al., 2016). Despite the clear benefits of the CRISPR/Cas9 approach in modulating miRNAs *in vivo*, the usual obstacles, such as off-target effects and lack of delivery vehicles with tissue specificity are yet to be overcome.

## LncRNA-Based Intervention Strategies

Given the fact that lncRNAs guide gene expression from start of transcription to protein translation, this class of molecules possess a promising therapeutic potential. The first study involving modulation of lncRNA expression for therapeutic purposes described the oncogenic lncRNA *H19*. In particular, it has been reported that *H19* is specifically expressed in over 30 tumours. Based on those findings, a plasmid expressing diphtheria toxin under control of the *H19* promoter (BC-819) has been intratumoraly injected into bladder tumour leading to a significant reduction in the tumour size in mice (Smaldone and Davies, 2010). This led to intiation of phases I and II human clinical trials, in which *H19* promoter-based plasmid is used to treat patients with different types of malignancies such as bladder, pancreatic, and ovarian cancers (Smaldone and Davies, 2010). Recently reported results seem promising, including in the treatment of early stage bladder cancer, BioCanCell report that BC-819 treatment resulted in 54% of patients recurrence-free at 24 months (Halachmi et al., 2018).

Currently, there are two main approaches to silence lncRNA expression, which are employed in pre-clinical models: the use of RNAi-based methods, such as siRNA, and LNA-GapmeR antisense oligonucleotides (ASOs), which induce RNase cleavage. Generally, the RNAi approach, including siRNA and short hairpin RNA (shRNA), which can be delivered via viral vector, is used predominanly for lncRNA that are localised in the cytoplasm. In particular, it has been demonstrated that siRNA targeting cytoplasmic lncRNA *SMILR* can reduce *SMILR* expression and attenuate pathological human saphenous vein SMC proliferation (Ballantyne et al., 2016). On the other hand, GapmeRs can be used for nuclear-localised lncRNA due to the fact that it induces degradation by RNase-H and is RISC-independent (Haemmig and Feinberg, 2017). Furthermore, GapmeRs can be used to modulate lncRNA expression for *in vivo*. In particular, intraperitoneal injection of GapmeRs in mice model of hindlimb ischaemia was able to significantly reduce *MALAT1* expression in the muscle tissue leading to poor blood flow recovery and diminished capillary density (Michalik et al., 2014). In another study, the GapmeR was used to inhibit the lncRNA *Chast*, which is upregulated in hypertrophic heart tissue from aortic stenosis patients. Notably, the *in vivo* delivery of GapmeR targeting *Chast* resulted in decrease in pathological cardiac remodelling with no side effects (Viereck et al., 2016). Finally, CRISPR/Cas9 genome editing method is an attractive emerging tool for modulation of gene expression including manipulation of lncRNAs. To date, the CRISPR-inhibition (CRISPRi) approach has been shown to knockdown the expression of six lncRNA including *GAS5*, *H19*, *MALAT1*, *NEAT1*, *TERC*, and *XIST* (Gilbert et al., 2014). Despite the fact that CRISPR/Cas9 method is at its nascent stages, coupled with viral-based delivery systems, it holds much potential in terms of ncRNA-based therapeutics.

## Conclusion

There remains little doubt that ncRNAs are important players in the pathology of vascular disease, and have been repeatedly demonstrated as key regulators of vascular biology in cell culture models, animal models, and in human samples. Aggressive lifestyle and risk factor management has had only a modest incremental effect on the trajectory of atherosclerotic disease in the last 70 years, even including the introduction of statins, and the huge improvements in surgical and interventional medicine. A new approach is now needed, and with the advent of CRISPR technology and the ongoing advances in delivery by viral vectors, non-coding gene therapy to up or downregulate culprit loci and transcripts has become a reality. With ever deeper understanding of molecular disease mechanisms and the refinement of safe and efficacious delivery methods the targeting of miRNA and lncRNA holds much promise for vascular disease treatment in future.

## Author Contributions

VM and JH were primary authors. JS, DN, and AB were involved in editing and reviewing of the manuscript.

## Conflict of Interest Statement

The authors declare that the research was conducted in the absence of any commercial or financial relationships that could be construed as a potential conflict of interest.
